# Predictive value of DNA methylation in the efficacy of chemotherapy for gastric cancer

**DOI:** 10.3389/fonc.2023.1238310

**Published:** 2023-09-12

**Authors:** Ye Li, Ning Mo, Dong Yang, QiuLu Lin, WenFeng Huang, Rensheng Wang

**Affiliations:** ^1^ Department of Oncology, The First Affiliated Hospital of Guangxi Medical University, Nanning, Guangxi, China; ^2^ Department of Radiation Oncology, the First Affiliated Hospital of Guangxi Medical University, Nanning, Guangxi, China; ^3^ Department of Oncology, The First Affiliated Hospital, Hengyang Medical School, University of South China, Hengyang, Hunan, China; ^4^ Department of Oncology, The Second Affiliated Hospital of Guangxi Medical University, Nanning, Guangxi, China

**Keywords:** gastric cancer, DNA Methylation, chemotherapy, survival, nomogram model

## Abstract

**Background:**

Gastric cancer (GC) is one of the most common causes of cancer-related death. Drug resistance in chemotherapy often occurs in patients with GC, leading to tumor recurrence and poor survival. DNA methylation is closely related to the development of cancer.

**Methods:**

To investigate the role of DNA methylation in chemotherapy resistance in GC patients, we conducted a comprehensive analysis using DNA methylation data and survival information obtained from The Cancer Genome Atlas. Univariate Cox analysis was performed to screen for differential DNA methylation of chemotherapy response in patients who did and did not receive chemotherapy. Multivariate Cox analysis was then performed to identify the independent prognostic genes. Gene Ontology and Kyoto Encyclopedia of Genes and Genomes pathway enrichment analyses were used to explore the biological function of the signature genes.

**Results:**

Patients receiving adjuvant chemotherapy for GC survived longer. 308 differentially methylated genes were demonstrated to be associated with prognosis. Six genes were optimally chosed for establisehing the risk model, including C6orf222, CCNL1, CREBZF, GCKR, TFCP2, and VIPR2. It was constructed based on the DNA methylation levels of these six genes: risk score = 0.47123374*C6orf222 + 9.53554803*CCNL1 + 10.40234138* CREBZF + 0.07611856* GCKR + 18.87661557*TFCP2 − 0.46396254* VIPR2. According to the risk score, patients receiving chemotherapy were divided into high- and low-risk groups, and the prognosis of the two groups was compared. The high-risk group had a shorter survival; however, this association was not present in patients without chemotherapy. The accuracy and predictive efficacy of the risk score in predicting the 1-, 3-, and 5-year survival of patients was evaluated with the receiver operating characteristic curve. In patients receiving chemotherapy, the area under the curve of the risk score for 1-, 3-, and 5-year survival was 0.841, 0.72, and 0.734, respectively. In patients who did not receive chemotherapy, the area under the curve was 0.406, 0.585, and 0.585, respectively. A nomogram model was constructed based on the risk score and clinical indicators. The model showed good consistency in the predicted probabilities and actual probabilities. Gene Ontology functional enrichment of these candidate methylated genes showed the following molecular functions: RNA binding, protein binding, mRNA binding, and nucleic acid binding; that they were mediated mainly through the following cell components: nuclear speck, nucleoplasm, nucleus, catalytic step 2 spliceosome, and the transcription factor AP-1 complex; and that they were involved in the following biological processes: mRNA processing, mRNA splicing, and RNA polymerase II promoter transcription. The Kyoto Encyclopedia of Genes and Genomes pathway enrichment results revealed that the signaling pathways mainly enriched were transcriptional misregulation in cancer, spliceosome, and the IL-17 signaling pathway.

**Conclusion:**

Our work identifies a six DNA methylated expression signature as a promising biomarker of chemo-resistance in GC, which provides new insights into the development of new strategies to overcome chemo-resistance in GC.

## 1 Introduction

Gastric cancer (GC), which is one of the most common gastrointestinal malignancies, is ranked fifth regarding the incidence rate among human malignancies and ranks third in mortality (1). Although the most effective method for the treatment of GC is surgical resection, most patients ignore the early symptoms until they feel obvious discomfort, resulting in many patients developing advanced GC, missing the best treatment opportunity. The postoperative 5-year survival rate of patients with early GC ranges from 60% to 80%. However, with disease progression, the 5-year survival rate decreases to 18% to 50%, in addition to the invasion of tumor cells into deeper tissues (e.g., the serosa and the muscularis) (2).

Chemotherapy drugs disseminate throughout most organs and tissues of the body. Hence, the main therapy method for advanced GC patients is chemotherapy (3). First-line chemotherapy drugs for GC mainly include 5-fluorouracil (5-FU), oxaliplatin, irinotecan, docetaxel, paclitaxel, and tegafur-gimeracil-oteracil potassium (S-1), which cause a high rate of tumor shrinkage in the clinic (4). However, the clinical outcomes of patients with GC receiving adjuvant chemotherapy are still significantly different due to the great difference in tumor sensitivity to chemotherapy drugs (5). Therefore, predicting treatment response is a highly important and clinically relevant issue in the hope to further improve chemotherapy outcomes in patients with GC.

DNA methylation is a form of DNA chemical modification that combines a methyl group with the cytosine 5-carbon position covalent bond of the CpG dinucleotide under the action of DNA methyltransferase, which changes the genetic expression without changing the DNA sequence (6). A large number of studies have shown that DNA methylation causes changes in the chromatin structure, DNA conformation, DNA stability, and the way that DNA interacts with proteins, thus controlling gene expression (7–11). The metabolism of various cells in the normal body is regulated by a variety of genes, among which the proto-oncogenes and anticancer genes are closely related to tumor cells. Anticancer genes play an important role in the cell cycle, DNA damage repair, cell differentiation, etc. (12). Epigenetic abnormalities in cancer include genome-wide hypomethylation and site-specific hypermethylation. Hypermethylation at the CpG island in the regulatory region of anticancer genes is one of the earliest and most frequent changes during tumorigenesis and is associated with transcriptional inhibition (13). Abnormal DNA methylation mainly occurs in the CpG-rich promoter region; abnormal hypermethylation in this region will prevent transcription factors from binding to promoters, thus preventing the transcription of anticancer genes or reducing the transcription expression levels (14). The silencing of anticancer genes may easily lead to out-of-control GC cell growth and proliferation, even increasing the chances of invasion to the periphery from the *in-situ* cell matrix, thus promoting the further development of GC (15).

In recent years, multiple studies have suggested a key role of abnormal methylation of certain genes in the occurrence and development of GC. DNA methylation analysis could provide information for early screening, efficacy, and prognostic assessment of patients with GC (16, 17) and other types of cancer (18). Patients with widespread gene hypermethylation show a lower overall survival rate, suggesting a correlation between gene methylation and chemotherapy sensitivity (18, 19). Fortunately, abnormal DNA methylation is a reversible epigenetic change, which opens new possibilities for improving the clinical efficacy of chemotherapy, namely, developing and searching for small molecule compounds that can change the state of DNA methylation and combine them with traditional chemotherapy drugs to bring hope for clinical treatment (20).

We speculate that the difference in the expression pattern of molecular biomarkers may be the cause of the change in prognosis of chemotherapy patients. Therefore, our study aims to use a comprehensive approach to identify and validate DNA methylation biomarkers that predict chemotherapy response in patients with GC. We performed an in-depth analysis of DNA methylation expression using data from The Cancer Genome Atlas (TCGA). The risk score model we built was based on a six-gene signature for the prediction of the adjuvant chemotherapy response in patients with GC and showed a better predictive value than the clinical indicators.

## 2 Materials and methods

### 2.1 Patients and clinical data

A total of 384 patients with GC p were included in the TCGA GC cohort (Cancer Genome Atlas Research et al., 2013). DNA methylation and mRNA expression datasets of TCGA GC cohort were obtained by https://portal.gdc.cancer.gov/. Clinical and chemotherapy information was downloaded from the TCGA dataset, including patients who did and did not receive adjuvant chemotherapy. The downloaded data is in full compliance with TCGA’s data access policy. All analyses were conducted in accordance with relevant guidelines and regulations.

### 2.2 Identification of specific prognostic-related methylated genes

To identify prognostic-related methylated genes in TCGA GC cohort, we first counted genes with significant negative correlations (Pearson correlation analyses) between DNA methylation levels and mRNA expression on a genome-wide scale (P < 0.05 and R < -0.3). Based on the genes that were screened, patients who received adjuvant chemotherapy were divided into high-expression and low-expression groups according to the median level of DNA methylation. Univariate Cox proportional hazard regression analysis was performed for each gene (P < 0.05). The same analysis was also performed in patients who did not receive adjuvant chemotherapy. Finally, multivariate Cox proportional risk regression analysis was used to construct the prediction models for methylated genes with significant prognostic value in the two groups of patients (variables with P < 0.05 were remained for final model construction).

### 2.3 Prognostic characteristics and nomogram construction

The least absolute shrinkage and selection operator (LASSO) is a method used for parameter selecting. High-dimensional regression variables are managed by shrinking all regression coefficients and forcing many variables to be completely zero without prior feature selection. The optimal DNA methylation gene for construction of the prediction model was selected by LASSO. The 1-penalty regularization parameter is determined by 10-fold cross validation using the R package “glmnet”. Based on DNA methylation expression of the coefficient weighted generated by LASSO penalty regression, a six DNA-methylated signature was identified with a lambda that minimized the partial likelihood bias. A risk score was calculated for each patient: score = L1·Exp1+ L2·Exp2 +…+ Ln·Expn. Expi represents the expression level of DNA methylation, Li, expressed as the LASSO coefficient. The time-dependent receiver operating characteristic (ROC) curve was used to evaluate the prediction accuracy of prognostic-related characteristics using the R package “survivvalroc”. The clinical parameters and the six DNA-methylation prognostic risk score of patients with GC receiving adjuvant chemotherapy was evaluated by the nomogram using the R package “rms”.

### 2.4 Functional enrichment analysis

Gene Ontology enrichment and Kyoto Encyclopedia of Genes and Genomes (KEGG) signaling pathway enrichment analyses for the low methylation/high expression of target genes were performed by DAVID (https://david.ncifcrf.gov/) (21). The bioinformatics online drawing tool (http://www.bioinformatics.com.cn/) was used for visualization.

### 2.5 Statistical analysis

The chi-square test was used to compare clinical features between patients with GC who were treated with and without chemotherapy. The correlation between the DNA methylation level and mRNA expression level was assessed by Pearson’s correlation coefficient. The univariate proportional hazard regression analysis was applied to determine independent prognostic variables for overall survival (OS). The Kaplan–Meier method and log-rank test were carried out to generate and compare survival curves. ROC curves were used to assess the predictive accuracy and sensitivity of each variable and the six DNA methylation signatures. The consistency between the predictive the actual results were evaluated by calibration curves. *P* < 0.05 was regarded as a statistically significant. The statistical analysis was performed using R 4.1.2.

## 3 Results

### 3.1 Clinical characteristics of GC patients receiving chemotherapy

**Figure 1:**
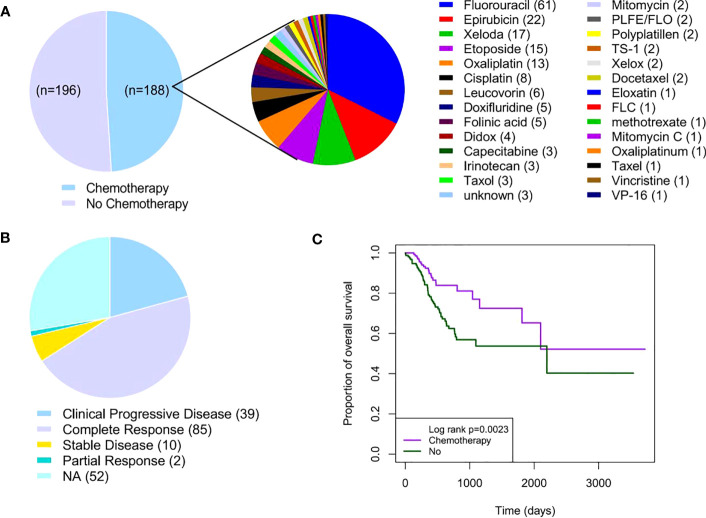
Adjuvant chemotherapy in patients with GC. **(A)** Distribution of adjuvant chemotherapy in TCGA GC cohort (n = 384). **(B)** Distribution of chemotherapy response in GC patients receiving adjuvant chemotherapy (n = 188). **(C)** Kaplan–Meier survival curves of GC patients with or without adjuvant chemotherapy. GC, gastric cancer; TCGA, The Cancer Genome Atlas.

A total of 384 GC patients were included in the TCGA GC cohort. Of the 384 GC patients, 48.96% (188/384) received adjuvant chemotherapy, whereas 51.04% (196/384) did not receive any type of chemotherapy (**Figure 1A**). The clinical and pathological characteristics are shown in **Table 1**. There was no statistical significance in gender, primary lymph node, pathological T and M stages, and histological grades between patients with and without chemotherapy (*P* > 0.05). Fluorouracil (61/188,32.45%) was the most commonly used chemotherapy drug in GC patients receiving chemotherapy. Nearly half of GC patients who received chemotherapy benefited from chemotherapy (**Figure 1B**). Notably, 45.21% of GC patients showed complete response to adjuvant chemotherapy (**Figure 1B**). The Kaplan–Meier survival curve showed that the hazard ratio of OS was 2.10 for patients who did not receive adjuvant chemotherapy (95% confidence interval: 1.34–3.29, *P* = 0.002) (**Figure 1C**). In conclusion, these results suggest that adjuvant chemotherapy is a viable treatment strategy for patients with GC.

**Table 1:** Clinical characteristics of patients.

Variables	Receiving chemotherapy (n = 188)		Not receiving chemotherapy (n = 196)			*P*
	n	%	n	%		
Gender						
Female	60	31.91	78	39.80	2.589	0.108
Male	128	68.09	118	60.20		
Age, years						
≤65	107	56.91	74	37.76	17.435	<0.001
>65	77	40.96	121	61.73		
NA	4	2.13	1	0.51		
Primary lymph node presentation						
No	14	7.45	12	6.12	5.460	0.065
Yes	173	92.02	176	89.80		
NA	1	0.53	8	4.08		
Pathologic T stage						
T1-2	42	22.34	59	30.10	2.982	0.084
T3-4	146	77.66	137	69.90		
Pathologic N stage						
N0-1	99	52.66	126	64.29	5.346	0.021
N2-3	89	47.34	70	35.71		
Pathologic M stage						
M0	169	89.89	180	91.84	0.437	0.508
M1	19	10.11	16	8.16		
Pathologic stage						
Stage I-II	70	37.23	105	53.57	12.724	0.002
Stage III-IV	110	58.51	79	4.0.31		
NA	8	2.66	12	6.12		
Histological grade						
G1-2	71	37.77	75	38.27	0.010	0.920
G3	117	62.23	121	61.73		

### 3.2 Identification of DNA methylation-associated prognostic biomarkers for GC patients receiving chemotherapy

Given the significant effect of chemotherapy in patients with GC, we wanted to determine whether there are prognostic biomarkers for GC in patients receiving adjuvant chemotherapy. As an important epigenetic modification, DNA methylation has shown good performance in cancer diagnosis and prognosis. We obtained DNA methylation expression data from the TCGA GC cohort. DNA methylation is the introduction of methyl groups into DNA molecules; this does not change the sequence of genes, but changes the activity of DNA segments.

**Figure 2:**
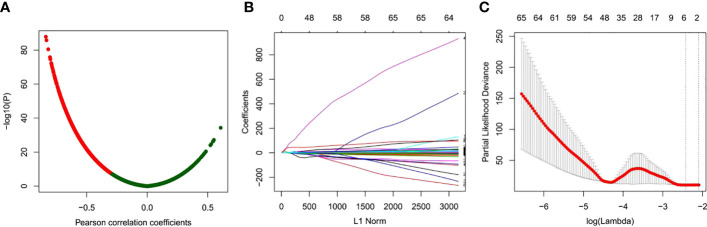
Identification of prognostic-related DNA methylation markers using LASSO regression models. **(A)** Correlation analysis between DNA methylation and mRNA expression. **(B)** Coefficient curve of clinical features, x-axis: L1 norm, y-axis: variable coefficient. The color of each line represents each candidate DNA methylation marker. **(C)** Ten-fold cross validation of LASSO regression for selecting the most appropriate clinical features. Each point represents a lambda value and error line, providing a confidence interval for the error rate of cross validation. The size of each model is given at the top of the figure. The vertical dashed line represents the value with the least error and the maximum lambda, where the deviation is within 1 SE of the minimum.

To search for DNA methylation prognostic markers that can be used as adjuvant chemotherapy for GC, we first identified the genes with a negative correlation between the DNA methylation level and mRNA expression on a genome-wide scale. DNA methylation has a multifaceted role in gene expression regulation, and it is not solely inhibitory. However, a significant portion of its regulatory effect is in a negative regulatory manner (**Figure 2A**). This analysis results in a total of 3,505 genes showing a negative correlation (**Figure 2A**).

Then, univariate Cox analysis was performed for each of the 3,505 candidates in GC patients with or without chemotherapy, respectively. Those genes exhibited prognostic value in GC patients received chemotherapy while having no indicative value for GC patients without chemotherapy were kept for further analyses. A total of 308 genes were identified (**Table S1**). Next, LASSO Cox regression analysis was performed for the 308 candidates to determine robust markers. Some coefficients were reduced to zero by the sum of the absolute values of forcing regression coefficients at a fixed value and the strongest prognostic marker was identified as the relative regression coefficient. Cross validation was used to prevent overfitting of the LASSO Cox model (**Figures 2B, C**). Finally, we obtained a six-DNA methylated gene signature.

**Figure 3:**
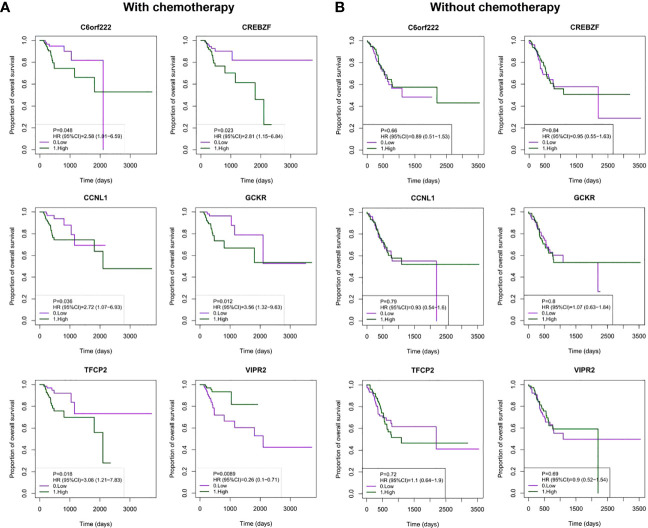
Kaplan–Meier analysis of OS with the DNA methylated signature in patients with or without chemotherapy. **(A)** Patients were divided into high- and low-methylation groups based on median DNA methylation levels, and survival analyses were performed for patients receiving chemotherapy. The patients were grouped (N_high_ = 85; N_low_ = 85) by the median value of the gene methylation levels. **(B)** Patients were divided into high- and low-methylation groups based on median DNA methylation levels, and survival analysis was performed in patients who did not receive chemotherapy. The patients were grouped (N_high_ = 82; N_low_ = 82) by the median value of the gene methylation levels. OS, overall survival.

The six-gene signature included C6orf222, cyclin L (CCNL1), CREB/ATF bZIP transcription factor (CREBZF), glucose kinase regulator (GCKR), transcription factor CP2 (TFCP2), and vasoactive intestinal peptide receptor 2 (VIPR2). To confirm the specific prognostic value of these six DNA methylation genes in GC patients receiving adjuvant chemotherapy, we examined the association between DNA methylation and OS in patients receiving and not receiving chemotherapy. As expected, the Kaplan–Meier survival curves showed that high methylation levels of C6orf222, CCNL1, CREBZF, GCKR, and TFCP2 were associated with poorer survival in patients receiving chemotherapy. In addition, high methylation levels of VIPR2 were associated with better survival in patients receiving chemotherapy (**Figure 3A**). In contrast, they had no prognostic value in patients who did not receive chemotherapy (**Figure 3B**). These results suggest that these genes may not have significant tumor biological functions in quiescent gastric cancer cells; however, when cells are exposed to chemical drug treatment, these genes may participate in regulating chemotherapy sensitivity by modulating cellular stress responses.

### 3.3 Prognostic value of 6-gene risk model for GC chemotherapy

**Figure 4:**
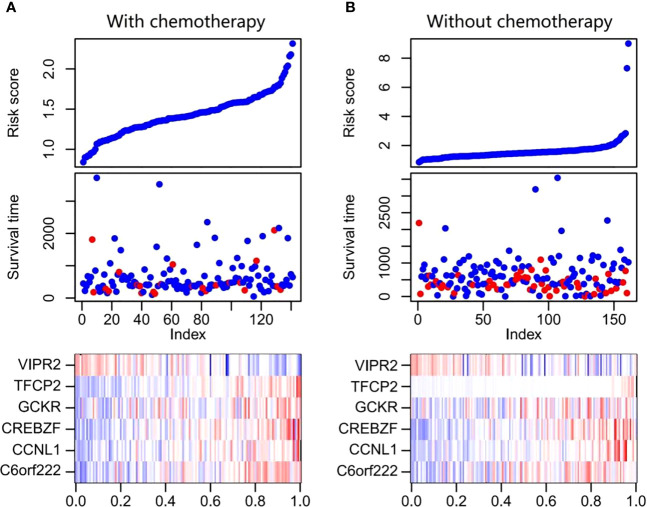
Risk score distribution, survival status, and DNA methylation expression profile of patients with GC. **(A)** The figure at the top shows the distribution of the risk scores for each patient receiving chemotherapy, the middle figure displays the survival status of each patient receiving chemotherapy, and the bottom figure is heat maps of the six DNA methylation signature. **(B)** The figure at the top shows the distribution of risk scores for each patient without chemotherapy, the figure in the middle shows the survival status of each patient without chemotherapy, and the bottom figure shows heat maps of the six DNA methylation signature. GC, gastric cancer.

To evaluate the prognostic value of methylation of C6orf222, CCNL1, CREBZF, GCKR, TFCP2, and VIPR2, we constructed a multivariate Cox regression model. The risk scoring formula is as follows: risk score = 0.47123374*C6orf222 + 9.53554803* CCNL1 + 10.40234138* CREBZF + 0.07611856* GCKR + 18.87661557*TFCP2 − 0.46396254*VIPR2. We used the above formula to calculate the risk scores of patients receiving and not receiving chemotherapy. The ranking of the risk scores of patients in each sample set is shown in **Figures 4A, B**. We found similar risk scores and DNA methylation expression in the two groups, suggesting that the signature was present before treatment and was not a result of chemotherapy. Among the six DNA methylation genes, C6orf222, CCNL1, CREBZF, GCKR, and TFCP2 were risk factors, and VIPR2 was a protective factor.

**Figure 5:**
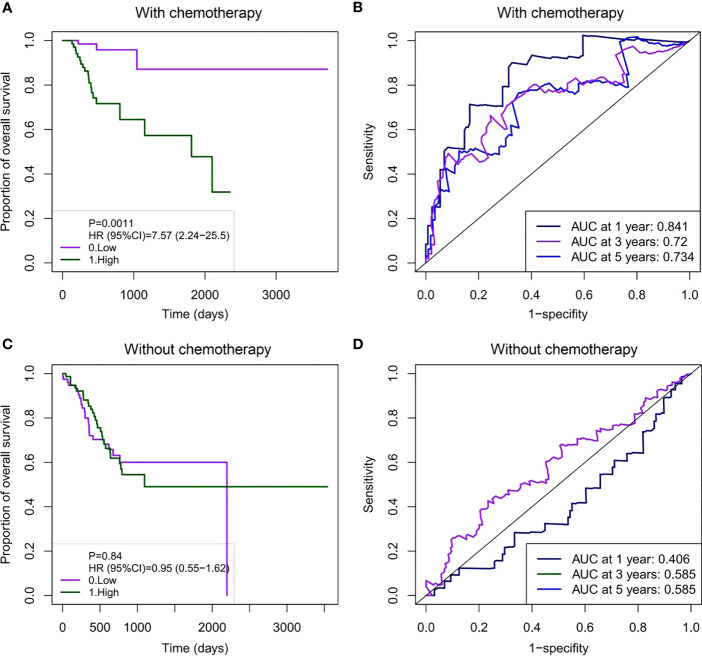
Survival analysis and time-dependent ROC curve based on the six DNA methylation signature risk scores in GC patients with or without chemotherapy. **(A)** Kaplan–Meier survival curve for the prognostic characteristics of risk scores in patients receiving chemotherapy. **(B)** Time-dependent ROC curves were used to assess the prediction accuracy of OS at different follow-up times (1-, 3-, and 5-year survival). **(C)** Kaplan–Meier survival curve for prognostic features of the risk scores in patients not receiving chemotherapy. **(D)** A time-dependent ROC curve was used to assess the prediction accuracy of OS at different follow-up times (1-, 3-, and 5-year survival). There were no deaths at 3–5 years (1095–1825 days) in GC patients not receiving chemotherapy. Thus, the AUC for the 3- and 5-year survival is the same. ROC, receiver operating characteristic; GC, gastric cancer; OS, overall survival; AUC, area under the curve.

We further evaluated the prognostic value of the model. Patients in the receiving and non-receiving chemotherapy groups were divided into high-risk and low-risk groups based on the median risk scores, respectively. Kaplan–Meier analysis showed that OS in the high-risk group was significantly worse than that in the low-risk group in patients receiving chemotherapy (**Figure 5A**). We used a time-dependent ROC analysis to assess the prognostic significance of risk scores for the six DNA methylated gene signature. The area under the ROC curves (AUCs) for the 1-, 3-, and 5-year OS in patients receiving chemotherapy were 0.841, 0.72, and 0.734, respectively, showing good prognostic value in predicting the outcome of chemotherapy (**Figure 5B**). However, Kaplan–Meier analysis of patients without chemotherapy showed no significant correlation between the risk scores and OS (**Figure 5C**). Consistently, the AUC for the 1-, 3-, and 5-year OS in patients without chemotherapy were 0.406, 0.585, and 0.585, respectively, indicating poor performance in patients without chemotherapy (**Figure 5D**).

**Figure 6:**
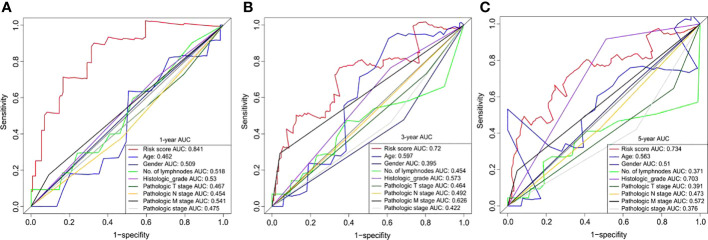
Time-dependent ROC curves for OS prediction based on the six DNA methylation signature risk scores and clinicopathological risk factors at different follow-up times (1-, 3-, and 5-year survival, respectively). ROC, receiver operating characteristic; OS, overall survival.

We also compared the prognostic value of the risk scores for the six DNA methylated genes with other clinical variables for chemotherapy outcomes in patients with GC. Clinical variables used for comparison included age, gender, number of lymph nodes, histological grades, pathological T, N, and M stages, and concomitant pathology stage. The results showed that the six DNA methylation gene signature risk scores outperformed all clinical variables, particularly in the short-term (1-year survival prediction) (**Figure 6A**). For 1- and 3-year survival predictions, the AUC was greater than 0.7 without clinical variables (**Figures 6A, B**). For long-term survival prediction, the risk score and histological grade showed a relatively better performance, with an AUC of 0.703 (**Figure 6C**). The time-dependent ROC analyses showed that all predictors, whether based on the six DNA methylation signature risk scores or other clinical variables, performed better in short-term survival than in long-term survival. Notably, the six DNA methylation signature risk scores outperformed other clinical variables. These results also demonstrate the clear advantages of molecular biomarkers in clinical settings.

### 3.4 Construction of a nomogram to predict the prognosis of GC patients after chemotherapy

**Figure 7:**
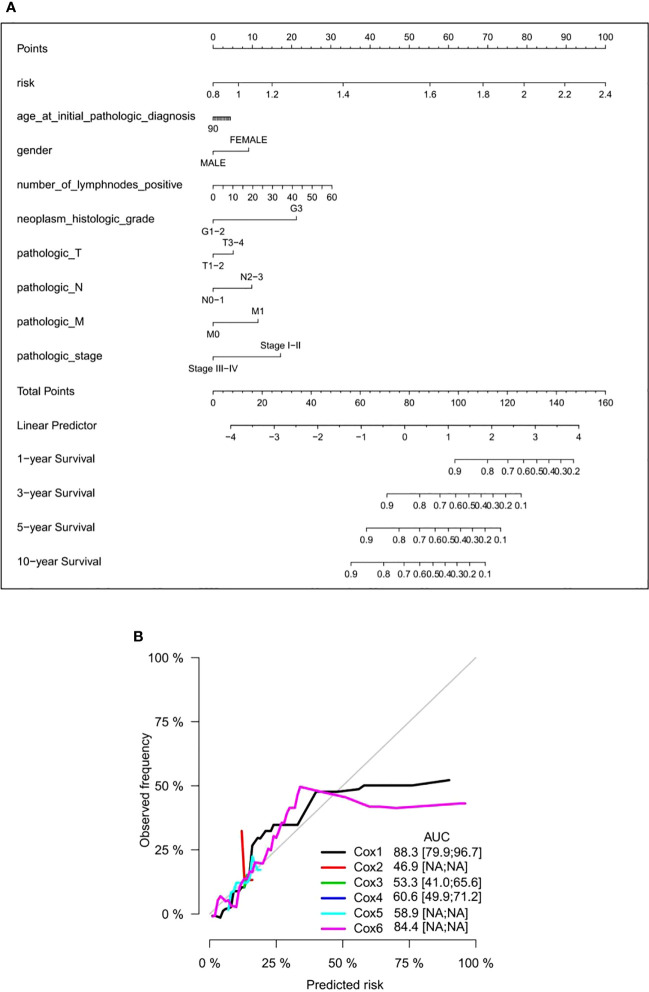
Establishment of a nomogram in GC patients receiving adjuvant chemotherapy. **(A)** Prediction of a nomogram in GC patients receiving adjuvant chemotherapy. There are nine components in this nomogram: the six DNA methylation risk score and eight clinicopathological variables. Each generates points based on a straight line drawn upward, and the total number of the three components of an individual patient is located on the “total points” axis; they correspond to the probabilities of 1-, 3-, 5-, and 10-year OS plotted on the two axes below. **(B)** Calibration curves, proportional risk model, clinical variable model, and combined nomogram of the six DNA methylation risk scores. AUC scores are expressed as point estimates. Cox1: risk score; Cox2: age; Cox3: gender; Cox4: histological grade; Cox5: age + sex + histological grade. Cox6: risk score + age + sex + histological grade. GC, gastric cancer; OS, overall survival; AUC, area under the curve.

To provide a clinical-related quantitative method for predicting the probability of 1-, 3-, 5-, and 10-year OS in GC patients receiving adjuvant chemotherapy, a prognostic nomogram was established with clinical variables including age, gender, number of lymph nodes, histological grade, pathological T, N, and M stages, concomitant pathology stage, and the six DNA methylated gene signature (**Figure 7A**). The OS nomogram calibration curve of GC patients receiving adjuvant chemotherapy was in good agreement with the observed results (**Figure 7B**). Hence, we considered this nomogram can be a promising prognostic predictor with high probability.

### 3.5 Function linkage of the risk signature

**Figure 8:**
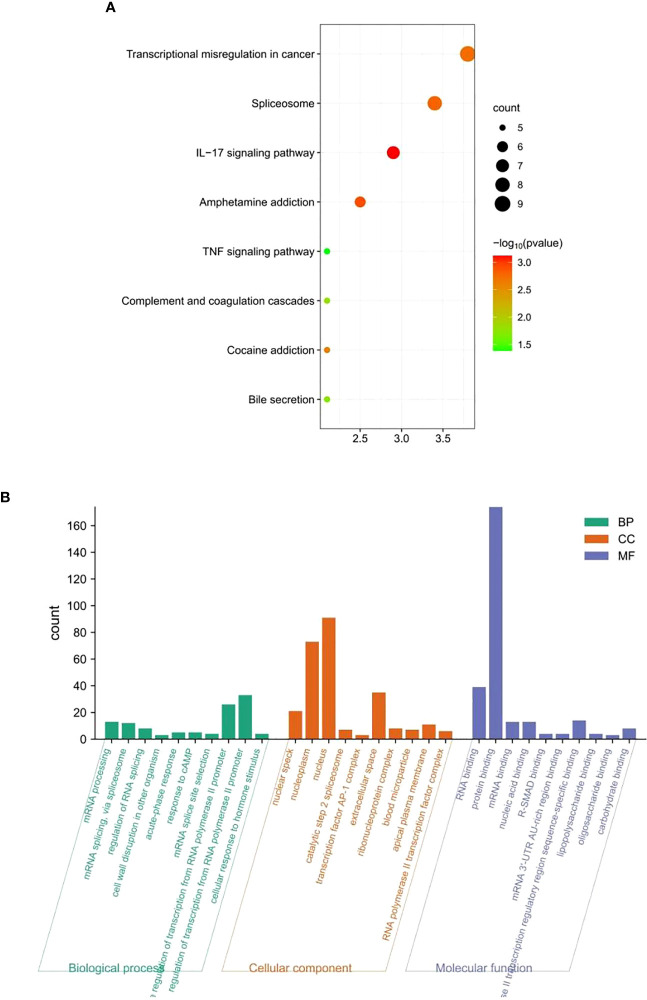
Functional enrichment analysis. **(A)** Kyoto Encyclopedia of Genes and Genomes and **(B)** Gene Ontology.

To explore the biological functions involved in these candidate genes, the STRING database was used to predict the proteins interacting with them (a total of 242 proteins; **Table S2**). Functional enrichment analysis of these interacting proteins was performed using the DAVID database and bioinformatics online mapping tool. KEGG results revealed that the signaling pathways mainly enriched were as follows: transcriptional misregulation in cancer, spliceosome, IL-17 signaling pathway, amphetamine addiction, TNF signaling pathway, complement and coagulation cascades, cocaine addiction, and bile secretion (**Figure 8A**). The Gene Ontology results are consistently enriched by RNA binding, catalytic step 2 spliceosome, mRNA processing, mRNA splicing, transcription factor AP-1 complex and RNA polymerase II promoter transcription (**Figure 8B**). Together, these results suggested mRNA metabolism, small molecular metabolism, and inflammatory and immune response are pivotal regulators in chemotherapy resistance of gastric cancer.

## 4 Discussion

Advanced GC patients are mainly treated with fluorouracil combined with other chemotherapy drugs; however, approximately 50% of patients with advanced GC are not sensitive to treatment (22). Meanwhile, it is still unclear which biomarkers can effectively predict chemotherapy sensitivity in patients with GC. Although a variety of biomarkers have been applied to predict fluorouracil sensitivity, including thymidylate synthase, thymidine phosphorylase, dihydropyrimidine dehydrogenase (DPD), human cytochrome P450, family 2, subfamily A, and polypeptide 6 (CYP2A6), their effectiveness is questionable (23, 24). Hence, the identification of biomarkers related to chemotherapy sensitivity remains an urgent task. Recent studies have found that abnormal methylation of genes may be involved in the occurrence and prognosis of GC (25). Therefore, this study aimed to identify a DNA methylation signature as a potential prognostic marker for patients with GC receiving adjuvant chemotherapy in the clinic.

In this study, we conducted a comprehensive analysis using DNA methylation data obtained from The Cancer Genome Atlas (TCGA). We developed a risk score feature based on six DNA methylation signatures to predict adjuvant chemotherapy response in patients with GC by using the multivariate Cox coefficient analyzed by LASSO, which was significantly superior to clinical variables. Based on the risk score, we also developed the nomogram model, which is a scoring system from 0 to 100 to predict 1-, 3-, 5-, and 10-year survival after adjuvant chemotherapy. Our results strongly suggest that each of the six signature and nomogram models of DNA methylation provides independent predictive values beyond traditional clinical variables. This approach allows for a detailed examination of DNA methylation patterns and their correlation with chemotherapy response and patient survival. Besides 5mC DNA methylation explored in this study, other types of DNA methylation have been found in genomic DNA from diverse species, e.g., N6-methyladenine (6mA) and N4-methylcytosine (4mC). Analyses of the genome-wide new types of DNA methylation paradigm may provide new biomarkers for predicting the prognosis of gastric cancer patients undergoing chemotherapy.

Through univariate and multivariate Cox analyses, the researchers identified six genes (C6orf222, CCNL1, CREBZF, GCKR, TFCP2, and VIPR2) that are differentially methylated and associated with prognosis in GC patients receiving chemotherapy. Among them, five ones (C6orf222, CCNL1, CREBZF, GCKR, and TFCP2) were risk factors for patients with GC, and the other one (VIPR2) was a protective factor. Notably, these DNA methylations had a specific predictive value for patients receiving chemotherapy, but not for those not receiving chemotherapy. Consequently, this type of DNA methylation may play a key role in the regulation of the chemotherapy response in patients with GC. C6orf222 is also known as BNIP5 (BCL2 interacting protein 5). The Bcl-2 gene (i.e., B-cell lymphoma/leukemia-2 gene) is an oncogene with an obvious inhibitory effect on cell apoptosis. Abnormal methylation of several apoptosis-related genes, such as DAPK and Bcl-2 interacting protein 3 (BNIP3), has been reported in various cancers (26), and BNIP3 methylation is associated with poor prognosis in GC (27). However, the effect of BNIP5 on tumor progression has not been reported, and this study demonstrated for the first time that BNIP5 methylation may also be related to chemotherapy response in GC.

CCNL1, a member of the cyclin family, interacts with CDK11A, CDK11B, CDK12, CDK13, and SFRS2 through its phosphorylated C-terminal domain. Amplification and overexpression of this gene have been reported in head and neck squamous cell carcinoma; the high level of amplification and prognostic overexpression is related to the degree of tumor differentiation, and the overexpression of this gene is increased in highly differentiated tumors (28). Previous analyses have suggested that CDK11 and cyclin L may be potential targets for cancer therapy (29).

The abnormal expression of CREBZF is closely correlated to cancer progression and prognosis; for example, high CREBZF expression predicts poor OS and/or progression-free survival in patient with ovarian cancer (30), and it is considered as a biomarker for the pathological progression of GC (31). However, the role of CREBZF methylation in GC has not been reported. Our study is the first to suggest that CREBZF methylation may also be associated with chemotherapy responses in GC.

GCKR encodes a protein belonging to the GCKR subfamily of the SIS (sugar isomerase) protein family. The gene is regarded as a candidate susceptibility gene for diabetes and non-alcoholic hepatitis (32). It was previously found that GCKR polymorphism may be an independent predictor of survival for metastatic GC patients receiving first-line EOF chemotherapy (epirubicin, oxaliplatin, and 5-FU combined chemotherapy) (33), which is consistent with the results of our work. GCKR methylation may also be associated with the chemotherapy response in GC.

TFCP2 is correlated with multiple cancers; it is a cancer-promoting factor for hepatocellular carcinoma, pancreatic cancer, and breast cancer, and can also be used as a tumor suppressor, such as inhibiting the growth of melanoma. In addition, TFCP2 also participates in epithelial–mesenchymal transformation and enhances angiogenesis (34). DNA hypermethylation promotes metastasis of colorectal cancer by regulating the binding of CEBPB and TFCP2 to CPEB1 promoters (35). Evidence for the association of TFCP2 methylation in GC has not yet been demonstrated, and we are the first to report that TFCP2 methylation may also be related to the chemotherapy response in GC.

Vasoactive intestinal peptide (VIP) is a gastrointestinal hormone in the pancreatotropin–VIP family. VIP affects the growth of some tumors, and VIP autocrine regulation is present in some cancers. In gastric adenocarcinoma tissues, the expression of VIP mRNA is upregulated, whereas that of VIPR mRNA is downregulated (36). Vega-Benedetti et al. (37) identified methylation changes in VIPR2 in colorectal cancer tissues, suggesting that VIPR2 might represent a new prognostic biomarker.

The DNA methyltransferase family consists of five members, namely DNMT1, DNMT2, DNMT3A, DNMT3B, and DNMT3L. Among them, only three have catalytic methyltransferase activity. Research has shown that DNA methyltransferases are closely associated with tumor drug resistance. Cancer stem cells (CSCs) are key factors in tumor resistance. So far, many small molecule inhibitors targeting DNA methylation have been developed. Among them, DNMT inhibitors such as azacitidine, decitabine and guadecitabine have entered clinical trials. Several studies have reported on the function and mechanism of DNA methylation inhibitors in chemotherapy for gastric cancer. A previous study demonstrated that the use of DNA methyltransferase inhibitors can reverse drug resistance in gastric cancer cells and restore their sensitivity to chemotherapy (38). Thus, the signature genes identified in this study may serve as pivotal targets of the DNMTs, inhibition of which by DNMT inhibitors will sensitize GC cells to chemotherapy.

Gene Ontology and Kyoto Encyclopedia of Genes and Genomes pathway enrichment analyses were performed to gain insights into the biological functions and signaling pathways associated with the identified methylated genes. This analysis provides valuable information about the molecular mechanisms involved in chemotherapy resistance in GC. KEGG results implied that the signaling pathways that were mainly enriched included transcriptional misregulation in cancer, spliceosome, IL-17 signaling pathway, amphetamine addiction, TNF signaling pathway, complement and coagulation cascades, cocaine addiction, and bile secretion. These results illustrate that the six DNA methylated gene signature may be involved in the chemotherapy response of GC through the above pathways. In the nucleus, the changes in DNA methylation and chromatin structure regulate gene transcription, and transcription dysregulation is a key feature of cancer. For example, Zhang et al. (39) indicated that mediator complex subunit 12 (MED12) is frequently mutated in benign tumors and cancers, and its abnormal expression is associated with the prognosis of various types of human cancers. The loss of function of MED12 is related to the development of resistance to chemotherapy drugs. Moreover, MED12 is modified by post-transcriptional regulation. Arginine methylation of MED12 has been validated to modulate MED12-mediated transcriptional regulation and response to chemotherapy drugs in human cancer cell lines (39). DNA methylation inhibits specific binding of the DNA binding protein (CTCF) to exons on DNA sequences and affects the splicing of exons. Some variable splicing products of genes that play a key role in tumor progression may have different functions and directly influence tumor progression and treatment. For example, BRCA1 usually has three splicing variants: BrCA1-full length containing all exons, Brca1-Delta11, which skips exon 11, and BrCA1-Delta11Q, with partial skipping of exon 11. BRCA1-Delta11q is significantly associated with breast cancer resistance to PARPi and cisplatin and patient survival, although inhibition of its splicing can improve sensitivity to PARPi (40); therefore, it has value as a therapeutic target. The survival, proliferation, and migration of cancer cells are directly promoted, and resistance to conventional chemotherapy drugs is induced through the IL-17RB/IL-17B signaling pathway (41). The KEGG pathway enrichment analysis suggested that this six DNA methylated signature may affect the response of chemotherapy drugs in GC patients through transcriptional regulation, the spliceosome, and the IL-17 signaling pathway. Gene Ontology analysis further explained that the six DNA methylation signature may be mediated through RNA binding, protein binding, mRNA binding, and nucleic acid binding in the nuclear speck, nucleoplasm, nucleus, catalytic step 2 spliceosome, transcription factor AP-1 complex and other cellular components, and, thus, participated in the biological processes of mRNA processing, mRNA splicing, RNA polymerase II promoter transcription, etc.

There are some limitations to our study. First, there was a lack of diversity in our patient cohort. Second, the predictive value of the six-gene signature model of DNA methylation needs to be further validated in a larger prospective cohort. Third, although we performed functional analyses of the six DNA methylation gene signature, further functional studies are needed.

In summary, the six newly identified DNA methylated genes proved to form an effective and stable model for predicting the prognosis of GC patients receiving adjuvant therapy, outperforming clinicopathological features. The clinical application of the six DNA methylated gene signature will assist in risk classification to guide personalized treatment of GC patients. Despite lacking systematic experimental validation, our study provides a basis for DNA methylation modules as a clinical tool for prognostic assessment after adjuvant chemotherapy. The six DNA methylated gene signature may also provide potential therapeutic targets for the treatment of drug resistance in GC patients.

## Data availability statement

The original contributions presented in the study are included in the article/**Supplementary Material**. Further inquiries can be directed to the corresponding authors.

## Author contributions

Conceived and designed the experiments: RW and WH. Performed the experiments: YL and NM. Analyzed the data: YL, NM, QL and DY. Wrote the paper: YL and WH. Critical review of manuscript: WH and RW.
